# The Contribution of Complement to the Pathogenesis of IgA Nephropathy: Are Complement-Targeted Therapies Moving from Rare Disorders to More Common Diseases?

**DOI:** 10.3390/jcm10204715

**Published:** 2021-10-14

**Authors:** Felix Poppelaars, Bernardo Faria, Wilhelm Schwaeble, Mohamed R. Daha

**Affiliations:** 1 Department of Internal Medicine, Division of Nephrology, University Medical Center Groningen, University of Groningen, 9700 AD Groningen, The Netherlands; faria_bernardo@yahoo.com (B.F.); M.R.Daha@lumc.nl (M.R.D.); 2 Nephrology and Infectious Disease R&D Group, INEB, Institute of Investigation and Innovation in Health (i3S), University of Porto, 4200-135 Porto, Portugal; 3 Department of Veterinary Medicine, University of Cambridge, Cambridge CB3 0ES, UK; hws24@cam.ac.uk; 4 Department of Nephrology, Leiden University Medical Center, University of Leiden, 2300 RC Leiden, The Netherlands

**Keywords:** complement, kidney, nephrology

## Abstract

Primary IgA nephropathy (IgAN) is a leading cause of chronic kidney disease and kidney failure for which there is no disease-specific treatment. However, this could change, since novel therapeutic approaches are currently being assessed in clinical trials, including complement-targeting therapies. An improved understanding of the role of the lectin and the alternative pathway of complement in the pathophysiology of IgAN has led to the development of these treatment strategies. Recently, in a phase 2 trial, treatment with a blocking antibody against mannose-binding protein-associated serine protease 2 (MASP-2, a crucial enzyme of the lectin pathway) was suggested to have a potential benefit for IgAN. Now in a phase 3 study, this MASP-2 inhibitor for the treatment of IgAN could mark the start of a new era of complement therapeutics where common diseases can be treated with these drugs. The clinical development of complement inhibitors requires a better understanding by physicians of the biology of complement, the pathogenic role of complement in IgAN, and complement-targeted therapies. The purpose of this review is to provide an overview of the role of complement in IgAN, including the recent discovery of new mechanisms of complement activation and opportunities for complement inhibitors as the treatment of IgAN.

## 1. Introduction to the Complement System

The complement system forms a major arm of innate immunity and is comprised of a large number of circulating and membrane-bound proteins [1]. The majority of these proteins circulate in an inactive form, but in response to pathogen-associated molecular patterns (PAMPs) and/or danger-associated molecular patterns (DAMPs), become activated through sequential enzymatic reactions [2,3]. Detection of these molecular patterns by the complement system is achieved via various pattern recognition molecules, and subsequent complement activation is realized by their associated serine proteases [4]. Complement activation can arise through three major pathways, including the classical pathway, the lectin pathway, and the alternative pathway, which all lead to the cleavage of C3, thereby forming C3a and C3b [5]. In the nomenclature of the complement system, when proteins are activated and cleaved into smaller fragments, the minor fragment is assigned the letter “a”, while the major fragment is assigned the letter “b”. The classical pathway recognizes immune complexes of IgM or hexameric IgG via C1q (the pattern recognition molecule of this pathway) together with the associated serine proteases C1r and C1s [6,7]. The lectin pathway contains six pattern recognition molecules: mannose-binding lectin (MBL), ficolin-1 (previously M-ficolin), ficolin-2 (previously L-ficolin), ficolin-3 (previously H-ficolin), collectin-10 (previously collectin liver 1), and collectin-11 (previously collectin kidney 1). These form a complex with MBL-associated serine proteases (MASPs) and recognize carbohydrate and acetylated structures on pathogens [8,9]. The alternative pathway continuously maintains low-level activity by the spontaneous hydrolysis of C3, called the ‘tick-over’, and thereby generates C3b, which can then covalently bind to various proteins, lipids, and carbohydrate structures on microbial surfaces [10]. Properdin has also been postulated to act as a pattern recognition molecule, thereby initiating alternative pathway activation [11,12], although these findings have not been consistent among studies and experimental conditions [13]. Besides PAMPs, complement activation is also brought about by DAMPs, e.g., activation of the classical pathway by C-reactive protein (CRP) or pentraxin-3 [14,15]. Other examples are the activation of the lectin pathway by L-fucose on stressed cells and cleavage of C3 by the neutrophil enzymes elastase or myeloperoxidase (MPO), resulting in alternative pathway activation [12,16,17].

Regardless of the pathway, progressive C3 activation results in the formation of the C5-convertases. Correspondingly, the C5-convertases cleave C5 into C5a, an extremely potent inflammatory mediator, and C5b. C5b is the initiator of the terminal step, and, together with the components C6 through C9, assembles the membrane attack complex (MAC), also called C5b-9 [18]. Traditionally, the MAC was found to be formed on Gram-negative bacteria such as Neisseria meningitidis, leading to cell lysis. However, the MAC can also assemble on the surface of other pathogens, erythrocytes, or damaged host cells. Moreover, on host cells, the amount of C9 in the MAC determines the pore size and thereby the function, which ranges from pro-inflammatory effects to cell death [19]. Complement activation also leads to the generation of other effector molecules, such as opsonins (C4b, C4d, C3b, iC3b, and C3dg) and anaphylatoxins (C3a, C5a), which can interact with their respective complement receptors (complement receptors (CR), C3a receptors (C3aR) as well as C5a receptors (C5aR)). To better understand the complement system, it is important to realize that activation can take place in the blood, called the fluid phase, as well as on surfaces, called the solid phase. However, under normal conditions, this system is tightly controlled by regulators present in the blood (fluid-phase regulators) and on cell surfaces (solid-phase regulators) [20]. Examples of solid-phase regulators include membrane cofactor protein (CD46), decay acceleration factor (CD55), the C3b receptor CR1 (CD35), and membrane attack complex-inhibitory protein (CD59), which are widely expressed on human cells. On the other hand, C1-inhibitor, C4b-binding protein (C4bp), Factor H, and Factor I are major fluid-phase regulators present in the blood.

## 2. Novel Insights into an Old Defense System

**Figure 1:**
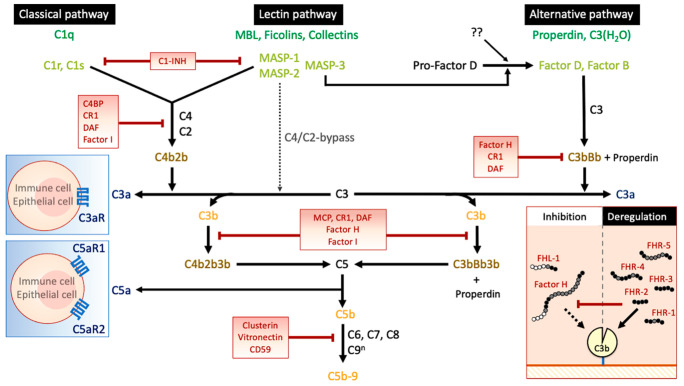
Overview of the complement system. Complement activation can be initiated via three different pathways: the classical pathway, the lectin pathway, and the alternative pathway. The classical pathway begins with the activation of C1, a complex composed of one C1q molecule (the pattern recognition molecule—dark green) as well as two C1r molecules and two C1s molecules (the serine proteases—light green). The lectin pathway begins via any of its pattern recognition molecules (dark green); that is, mannose-binding lectin (MBL), ficolins, or certain collectins, which work together with MBL-associated serine protease 1 (MASP-1) and 2 (MASP-2). Activation of either the classical or the lectin pathway leads to the cleavage of C4 and C2 and the formation of the C4bC2a complex, also known as the C3-convertase (gold). In the alternative pathway, activation occurs via the spontaneously thioester-hydrolyzed form of C3 (C3(H_2_O)) or via surface interactions of properdin (the pattern recognition molecules—dark green), which acts with Factor B and Factor D (the serine proteases—light green) to form the C3-convertase C3bBb (gold). Overall, all three pathways lead to the formation of their respective C3-convertases (gold), which in turn cleave C3 into C3a (an anaphylatoxin—blue) and the opsonin C3b (yellow). MASP-2 has also been shown to directly cleave native C3, thereby bypassing C2 and C4 in the activation of the lectin pathway; this is also known as the C4/C2 bypass mechanism (grey). Recently, MASP-3 was revealed to cleave pro-Factor D into Factor D, establishing a novel link between the lectin and alternative pathway. Although MASP-3 is responsible for the main activation of pro-Factor D, there is also an unknown alternative pro-Factor D activator. Increasing densities of C3b through activation of C3 by the C3-convertases favors the formation of the C5-convertases (gold). In the classical and lectin pathways, C5-convertase is formed by a complex of C3b with C4b and C2a known as C4b2b3b. In the alternative pathway, an additional C3b binds to the C3 convertase (C3bBb) to form the C5-convertase C3bBb3b. Properdin is a key positive regulator of complement activity which acts by stabilizing alternative pathway C3- and C5-convertases. The C5-convertases (C4b2b3b and/or C3bBb3b, respectively) cleave C5 to generate the potent chemoattractant C5a (an anaphylatoxin—blue) and C5b (yellow), the initial component of the membrane attack complex. Next, C6, C7, C8, and C9 bind serially to surface-bound C5b to form the final complex, C5b-9 (yellow). Further interactions with additional C9 molecules, up to 17 molecules, widens the inner pore of the membrane attack complex. In addition, the anaphylatoxins C3a and C5a bind to their respective receptor (blue), C3a-receptor (C3aR), C5a receptor 1 (C5aR1), and C5a receptor 2 (C5aR2) on target cells to mediate a variety of inflammatory responses. In parallel to these activation pathways, complement regulation is established through membrane-bound and soluble complement inhibitors. In the classical and lectin pathway, C1-inhibitor (C1-INH) regulates the activity of the pattern recognition molecules and associated serine proteases, whereas C4b-binding protein (C4BP) inhibits activation at the C4 level. Factor I and Factor H act on C3- and C5-convertases. In addition, the membrane-bound inhibitors complement receptor 1 (CR1/CD35) and membrane cofactor protein (MCP/CD46) act as co-factors for Factor I, whereas decay-accelerating factor (DAF/CD55) accelerates the decay of C3-convertases. The membrane-bound regulator CD59, as well as soluble regulators clusterin and vitronectin, impair the formation of C5b-9. The Factor H protein family consists of Factor H, Factor H-like protein 1 (FHL-1), and five Factor H-related proteins (FHR). Factor H consists of 20 domains. The first four domains (white) provide the inhibitory function of the protein, while the internal region (black) and the last two units (black) are needed for binding to cells and tissue sites. FHL-1 is composed of the first 7 domains of Factor H, whereas the FHRs have structural homology to binding domains (black) of Factor H. The current belief is therefore that FHRs compete with Factor H (and FHL-1) for binding to certain surfaces. The binding of Factor H (and FHL-1) will lead to complement inhibition, whereas binding of the FHRs will further enhance complement activation.

Today, our appreciation of the complement system has advanced immensely. As a result, it is easy to assume that its role has been completely unraveled. However, recent reports have identified novel players and unexpected functions of the complement system and have demonstrated that there is more to it than we now know. Important recent discoveries include: (i) the cross-talk between the lectin and alternative pathways, in particular by MASP-3; and (ii) the capacity of Factor H-related proteins (FHRs) to antagonize the ability of Factor H to regulate complement activation (Figure 1). These discoveries are important for a better understanding of the involvement of the complement system in IgAN. 

Although the complement system is presented as three separate and clearly outlined pathways, multiple reports have demonstrated that the pathways are closely connected and intertwined. Earlier studies demonstrated that initial complement activation by the classical pathway, as well as the lectin pathway, is amplified by the alternative pathway, and this amplification loop is estimated to contribute up to ~80% of the achieved complement activation [21,22]. Recently, the contrary has also been demonstrated, as the lectin pathway was shown to be indispensable for efficient alternative pathway activation [23]. In the lectin pathway, binding of MBL, ficolins, or collectins to their ligands leads to autoactivation of MASP-1, which thereafter activates MASP-2 [8]. Subsequently, MASP-2 cleaves C4, whereas C2 is cleaved by both MASPs, resulting in the formation of C3-convertases (i.e., C4bC2a) [24]. These convertases can then cleave C3 into C3a and C3b. Recently, another serine protease was discovered, namely MASP-3. This third serine protease is an alternative splicing product of the MASP-1 gene, and its functional significance remained an enigma until recently. In an elegant series of experiments, Dobo et al. revealed that activated MASP-3 cleaves pro-Factor D into Factor D, thereby establishing a crucial link between the lectin and the alternative pathway [25]. Using a specific MASP-3 inhibitor, they were able to block the conversion of pro-Factor D into Factor D. Additionally, Factor D isoforms were analyzed in MASP-1/3-deficient Malpuech–Michels–Mingarelli–Carnevale patients and MASP-1/3^−/−^ mice [26]. These experiments demonstrated that MASP-3 is responsible for the main activation of pro-Factor D, while also stressing that an alternative pro-Factor D activator exists [23]. In a follow-up study by the same authors, MASP-3 was shown to be mostly present as an active enzyme in blood under normal circumstances [27]. Proprotein convertase subtilisin/kexin 6 (PCSK6) was later identified as the main activator of MASP-3, thus completing the elucidation of this novel axis which is involved in the activation of the alternative pathway [28].

Dysregulation of the complement system is a causal factor in the development of various inflammatory and autoimmune diseases [29]. The complement regulatory protein Factor H is a key player in maintaining balance [30]. The discovery that Factor H consists of 20 units, known as “short consensus repeats” (SCR), has helped to attribute the different functions of Factor H to specific domains within the protein [31]. The first 4 units (SCRs 1–4) provide the inhibitory function of the protein, while the internal region (SCRs 6–8) and the last 2 units (SCRs 19, 20) are needed for binding to cells and tissue sites [32,33,34]. Genetic and acquired factors can cause distinct molecular defects in Factor H and can thereby give rise to different diseases [35]. For example, mutations that cause a complete Factor H deficiency lead to uncontrolled complement activation in the fluid phase and are linked to C3 glomerulopathy (C3G), a heterogeneous histopathological entity characterized by glomerular C3 deposition [36]. Heterozygous mutations in Factor H only lead to partial deficiencies, and these are associated with C3G but also with other diseases such as age-related macular degeneration (AMD), atypical hemolytic uremic syndrome (aHUS), and IgAN [37]. Alternatively, mutations or autoantibodies that affect the binding sites of Factor H give rise to aHUS because they impair the ability of Factor H to control complement activation on surfaces without modifying complement regulation in the fluid phase [38,39,40,41]. In addition to Factor H, humans also have five FHRs: FHR-1, FHR-2, FHR-3, FHR-4 and FHR-5 [42]. The genes for the FHRs are believed to have arisen during evolution through duplication events of the Factor H gene [43]. Subsequently, the FHRs have structural homology to Factor H, but they all lack the first four units of Factor H (i.e., the inhibitory region). Thus, based on their structure, FHRs were originally predicted to be irrelevant for maintaining immune homeostasis. However, recent work has opposed this notion. Genetic studies have revealed that variants of FHRs are strongly associated with human pathology, mostly those involving the kidney and retina [44]. These findings indicate that the FHRs could be involved in their pathophysiology. Nevertheless, the distinct molecular mechanisms by which FHRs contribute to disease are poorly understood. All FHRs are predicted to bind similar ligands as Factor H but lack its regulatory activity. The current belief, therefore, is that the FHRs antagonize the ability of Factor H to regulate complement activation [30,42]. Thus, FHRs act as de-regulators of the complement system by competing with Factor H for binding to surfaces that require protection. Notably, clear differences exist among the different FHRs [37]. For instance, FHR-1, FHR-2, and FHR-5 can dimerize to form homodimers. Conversely, FHR-3 and FHR-4 lack this dimerization motif in their N-terminal domains. Initial work proposed that, in addition to homodimers, heterodimers could also be formed between FHR-1 and FHR-2 as well as FHR-1 and FHR-5, whereas FHR-2/FHR-5 heterodimers would only occur if FHR-1 was absent [45]. However, recently, another study proposed that only four dimers are present in the blood: FHR-1, FHR-2, and FHR-5 homodimers, as well as heterodimers of FHR-1/FHR-2 [46]. Additional studies are thus needed to verify the compositions of these dimers in the circulation, together with the exact function of these dimers. Currently, these dimers are believed to have increased avidity for tissue-bound complement fragments, enabling them to more efficiently compete with Factor H [45].

## 3. The Unique Susceptibility of the Kidney to Complement-Mediated Injury

The complement system is more than a defense system against pathogens, as it also acts as a surveillance system to preserve tissue homeostasis and stimulate repair [4]. As a consequence, complement can be the initiator or aggravating factor in renal diseases. The complement system contributes to kidney disease via different mechanisms: excessive or inappropriate activation, insufficient regulation, or ineffective clearance [29]. Overwhelming activation can be triggered when the complement system is exposed to vast amounts of PAMPs or DAMPs, as seen in sepsis and brain death [47,48]. Separately, immune recognition of apparently innocent materials or biological surfaces can create inappropriate complement activation, as seen in hemodialysis and transplantation [49,50,51,52,53]. Independently, loss of complement regulation due to genetic alterations can lead to an imbalance that can cause tissue damage, as seen in C3G and aHUS [31,54]. Finally, ineffective removal of immune complexes and cellular debris due to deficiencies in complement components can induce autoimmune diseases such as lupus nephritis [31,55]. A combination of these mechanisms is also possible (e.g., initial insufficient regulation that leads to excessive activation), reflecting the complexity of complement-mediated renal diseases.

The kidney is particularly susceptible to complement-mediated injury, possibly due to the high blood flow, ultrafiltration, relatively low expression of complement receptors, and local variations in electrolyte concentrations and pH [56]. In addition, the local synthesis of complement proteins in the kidney seems to be of major significance [57,58]. The main source for complement factors is the liver, with the exception of C1q, properdin, and C7, predominantly produced by leukocytes, and Factor D synthesized by adipocytes [59,60,61,62,63,64]. However, accumulating evidence indicates that a wide range of cell types in the kidney are also able to produce complement components [58]. Renal tubular epithelial cells can produce virtually all complement proteins and are the main renal source of complement [65,66]. Under basal conditions, the kidney produces up to 5% of the circulating C3, but this can increase up to 16% during inflammation [67]. In renal diseases, complement activation can therefore occur in different compartments, namely systemically (i.e., in blood) or locally (i.e., in the kidney). Local production of complement proteins seems to be predominantly important at serum-restricted sites, such as the renal interstitium [68]. Local complement activation will lead to increased local vascular permeability, subsequently resulting in the leakage of systemic complement proteins and the initiation of the immune response [8]. Recently, a possible new compartment has been suggested, namely intracellular complement activation [69,70]. However, the occurrence of intracellular complement activation in renal disease and its relevance remains to be investigated. 

## 4. The Complement System in IgA Nephropathy

**Figure 2:**
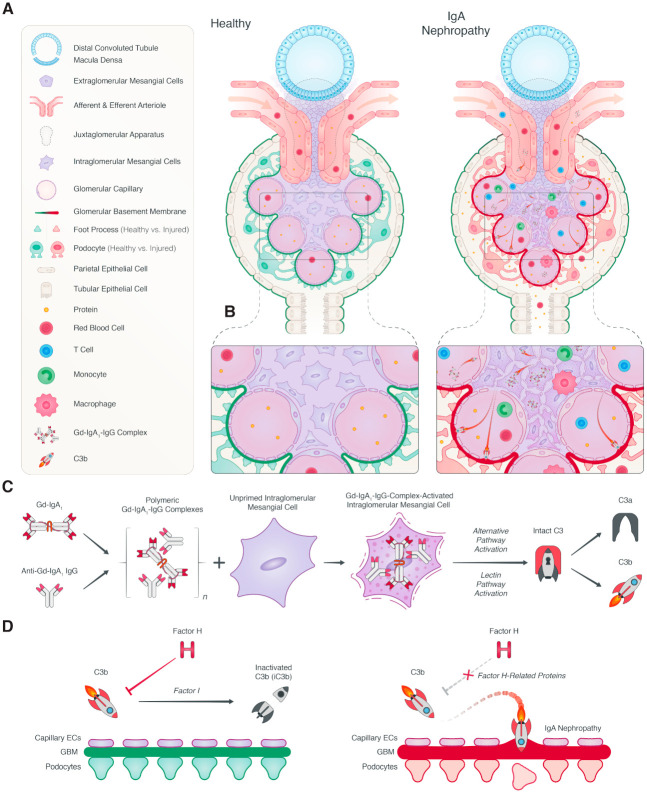
The role of complement activation in IgA nephropathy. (**A**) In a healthy glomerulus, filtration of blood occurs, and intact podocytes prevent the loss of proteins. In IgA nephropathy (IgAN), deposition occurs of immune complexes containing polymeric galactose-deficient IgA1 in the glomerular mesangium. (**B**) This leads to immune activation and induces proliferation of mesangial cells, increases the synthesis of extracellular matrix, and causes glomerular basement membrane (GBM) thickening, podocyte injury and protein loss. (**C**) Polymeric IgA1 and IgA1-containing immune complexes can activate both the alternative and lectin pathway, leading to the cleavage of intact C3, thereby forming C3a and C3b. (**D**) Factor H is a key regulator of the complement system, and together with Factor I, Factor H cleaves C3b to iC3b. Lastly, the Factor H-related proteins can compete with the regulatory functions of Factor H, thereby promoting complement activation.

IgAN is the most common form of glomerulonephritis and an important cause of kidney failure [71]. The diagnosis is confirmed by a kidney biopsy, revealing predominant deposition of IgA1 in the renal mesangium. IgAN is believed to have a multi-hit pathogenesis, namely: genetically determined high circulating levels of galactose-deficient IgA1, subsequent synthesis of antibodies directed against these galactose-deficient IgA, binding of these autoantibodies to IgA1 to form immune complexes, and finally, deposition of the immune complexes in the renal mesangium, leading to immune activation and renal damage [72]. The presence of complement activation in patients with IgAN was reported almost five decades ago [73]. However, the relevance of the complement system to the pathophysiology was not immediately recognized. Recent advances have increased our knowledge of the role of the complement system in the pathophysiology of IgAN (Figure 2). Additionally, these developments have enabled the development of novel therapeutic strategies for IgAN that are currently being tested in clinical trials.

### 4.1. Local Complement Activation

Very early on, in the initial reports about the disease, complement deposition was already described in renal biopsies of IgAN patients [73]. These first descriptions of the disease reported mesangial deposition of IgA and C3 in renal biopsies in more than 90% of cases. However, the importance of local complement deposition in IgAN was not recognized until later reports revealed that the extent of C3 deposits in the mesangium correlated with the severity and progression of IgAN [49,74,75,76,77]. In these recent studies, glomerular C3 deposition was observed in 71 to 100% of IgAN patients [78,79,80,81]. Next to glomerular IgA and C3 deposits, properdin and C5b-9 are almost always present, while C1q is typically absent [49,73,82,83,84]. Local complement activation in IgAN was therefore thought to result from the alternative pathway. In accordance, early studies demonstrated the ability of IgA to activate the alternative pathway in vitro [85,86]. The mechanism behind IgA-induced alternative pathway activation is poorly understood, but the polymerization of IgA is critical. Other proteins of the alternative pathway have also been identified in kidney biopsies of patients with IgAN, including Factor B, Factor H, and the FHRs [87,88,89,90,91,92,93]. Multiple studies have also investigated the utility of urinary Factor H levels for the assessment of disease activity and prognosis in patients with IgAN [89,93,94,95]. Surprisingly, urinary levels of Factor H were positively associated with markers of IgAN severity and disease progression. It is noteworthy to mention that because of the structural homology between Factor H and FHRs, it is very well possible that these Factor H assays also detected the FHRs and thereby confound the results [37]. Proteomic analysis of micro-dissected glomeruli in IgAN biopsies have verified the presence of Factor H, FHR-1, FHR-2, FHR-3, and FHR-5 [96]. Moreover, FHR-2 and FHR-5 were significantly more abundant in the glomeruli of patients with progressive IgAN compared to non-progressive IgAN. The presence of FHRs in IgAN was first mentioned 20 years ago by Murphy et al., who described glomerular FHR-5 deposits in a range of renal biopsy specimens including IgAN [97]. Mesangial deposition of FHR-5 was detected in all 20 IgAN cases, and the pattern of FHR-5 deposition was comparable, but not always identical, to that of IgA, C3, and sC5b-9. Recently, increased glomerular staining for FHR-5 was shown to be associated with progressive disease, while a trend was seen for greater FHR1 staining [88]. In contrast, glomerular Factor H staining was significantly reduced in patients with progressive IgAN in comparison to stable disease. Glomerular FHR5 deposition positively correlated with glomerular staining of C3 activation fragments, C5b-9, and absent Factor H staining. 

These results are in line with the hypothesis that FHRs compete with Factor H, thereby amplifying complement activation. No association was seen between glomerular staining for FHR-1 and IgAN severity. Similarly, a Chinese cohort found mesangial staining of FHR-5 in 57.1% of IgAN cases, and FHR-5 deposition was associated with histologic injury [98]. FHR-5 co-localized and correlated with IgA as well as C3 deposits. IgAN patients with endocapillary hypercellularity and segmental glomerulosclerosis had greater glomerular FHR-5 staining. Interestingly, the authors reported sex differences in glomerular FHR-5 depositions, with greater staining in male IgAN patients. These data indicate that FHR-5 might be a key contributor to complement dysregulation in IgAN (Table 1). It is important to mention that FHR-5 detection by immunohistochemistry in the study by Medjeral-Thomas et al. and by Guo et al. was achieved by using rabbit polyclonal antibodies against FHR-5 [88,98], creating the possibility of cross-reactivity with other FHRs [37].

Although previous studies had shown that the role of the classical pathway is limited in IgAN, little attention had initially been paid to the lectin pathway until the group of Fujita et al. demonstrated glomerular deposition of MBL and MASP-1 in IgAN which co-localized with C3b and C5b-9 deposits [107]. A follow-up study showed mesangial deposits of MBL, MASP-1, and C4 in over half of the IgAN cases, and also showed that IgA2 co-localized with MBL and MASP-1 in the mesangium of these patients [108]. Later, additional components of the lectin pathway, such as ficolin-2 deposition, were also demonstrated in IgAN [78,109]. In agreement with these results, IgA was shown to induce activation of the lectin pathway in vitro [16]. Interestingly, lectin pathway presence in renal biopsies is only seen in a subset of IgAN patients [78,107,108]. In the landmark paper by Roos et al., glomerular deposition of Ficolin-2 and MBL was shown to be associated with a higher level of histological damage, demonstrated by increased mesangial and extracapillary proliferation, interstitial infiltration, and glomerular sclerosis, as well as with heavier proteinuria [78]. Urine levels of MBL and C4d have also been shown to be associated with markers of disease activity and severity in IgAN, and urinary levels of these complement proteins correlate with their respective mesangial deposits [110,111]. These findings were further supported by the association of mesangial C4d deposition with disease progression and lower renal survival in IgAN patients [80,81,109,112]. Espinosa et al. was the first to demonstrate that mesangial C4d staining and absent C1q (indicative of lectin pathway activation) in IgAN patients was associated with progression to kidney failure [112]. In a follow-up study, they assessed the prognostic value of glomerular C4d staining in IgAN in a larger cohort [80]. Mesangial C4d deposits were identified in 39% of the 283 patients and C4d-positive staining was an independent risk factor for the development of kidney failure in IgAN. These results had important practical implications, because C4d staining is already routinely used in clinical practice for the diagnosis of antibody-mediated humoral rejection in biopsies from kidney transplant patients [113]. Various studies have subsequently investigated the use of C4d staining in IgAN as an indicator of disease severity and as a risk factor for kidney outcomes in different geographical populations, stages of chronic kidney disease, and degree of proteinuria [77,81,109,114]. Recently, a meta-analysis was performed on IgAN studies evaluating the relationship between glomerular C4d deposits and kidney outcomes, and the authors found that glomerular C4d deposition in IgAN was associated with higher histological disease activity, faster decline in eGFR, and kidney failure [115]. However, C4d deposition in IgAN is not limited to the glomeruli and has also been documented in the vasculature of the kidney. Arteriolar C4d deposits in IgAN are also associated with faster disease progression, and the association with progressive kidney disease was found to be stronger than glomerular C4d deposits [79]. In accordance, in IgAN, C3 deposition is also routinely found in extraglomerular areas such as in Bowman’s capsule and in the arterioles, and these C3 deposits also seem to be associated with worse outcome [116].

Glomerular C5b-9 deposition in IgAN was first reported over 3 decades ago by Rauterberg and colleagues [84]. Terminal pathway activation, as shown by C5b-9, was present in all IgAN cases, but not in controls. Furthermore, mesangial deposits of C5b-9 co-localized with both IgA and C3d deposition. Correspondingly, Medjeral-Thomas et al. reported that mesangial C5b-9 staining significantly correlated with both mesangial C3b/iC3b/C3c and C3d staining [117]. C5b-9 deposition in the glomeruli has been suggested to contribute to podocyte injury and subsequent proteinuria in IgAN [118]. Furthermore, decreased expression of CR1 (also known as CD35) on podocytes correlated with glomerular C5b-9 deposition in IgAN. These findings insinuate that reduced CR1 expression perhaps increases the sensitivity of podocytes to complement attack in IgAN [118]. However, decreased CR1 expression on podocytes is a shared histopathological feature among glomerular diseases and is not specific to IgAN [119]. In addition to the mesangium, C5b-9 can also be found along the capillary wall in the glomerulus, Bowman’s capsule, the tubular basement membrane, and the vascular wall [120]. In recent studies, the presence of C5b-9 in IgAN biopsies has been confirmed by proteomics analysis of microdissected glomeruli [96]. Terminal pathway components were significantly more abundant in IgAN biopsies than in healthy controls, as well as in IgAN cases with progressive disease compared to IgAN with non-progressive disease. Furthermore, terminal pathway components were associated with a higher histological score and lower kidney function. In accordance, multiple studies have found a relationship between C5b-9 staining in IgAN and histological lesions as well as clinical outcomes [120]. Overall, glomerular C5b-9 deposition in IgAN correlates with the extent of glomerulosclerosis, mesangial expansion, hypercellularity, interstitial inflammation, and fibrosis as well as tubular atrophy, whereas tubular C5b-9 staining is associated with the extent of tubular atrophy, interstitial inflammation, and interstitial fibrosis [121,122,123,124,125,126,127,128,129]. Regarding clinical outcome, glomerular and tubular staining of C5b-9 has been associated with kidney function, proteinuria, and progressive IgAN [84,117,124,125,127,130,131]. Correspondingly, increased C5aR1 expression has also been reported in renal biopsies of IgAN cases, and C5aR1 staining also correlates with histological injury, proteinuria, and kidney function [132]. C5aR1 staining in IgAN was mainly found on glomerular mesangial cells, tubular epithelial cells, and interstitial infiltrating cells. Similarly, urine levels of C5a and soluble C5b-9 (sC5b-9) have been found to be associated with markers of disease activity in IgAN, thereby further supporting the significance of the terminal pathway [93,132]. 

### 4.2. Systemic Complement Activation

In addition to local complement activation in IgAN, systemic complement activation has also been evaluated. Although plasma C3 levels are usually normal, activation fragments of C3 are elevated in some patients and correlate with the levels of IgA-containing immune complexes, histology, and disease progression [133,134,135,136]. However, most of these studies were performed in the 1980s and 1990s. Proteomics analysis of circulating deglycosylated IgA-immune complexes confirmed the presence of C3 activation fragments, such as iC3b, C3c, and C3dg [137]. More recently, systemic C3 levels were investigated in 343 IgAN patients [76]. Only 19% had serum C3 levels below the normal range. However, IgAN patients with decreased C3 levels had higher extents of mesangial C3 deposits in their renal biopsy than those with normal C3 levels. Furthermore, serum C3 levels were significantly associated with progression to kidney failure, but the predictive value of serum C3 was lower than clinical markers such as proteinuria and eGFR. In contrast, a separate study of 496 patients with IgAN, of whom 22% had low levels of C3, reported that serum C3 levels did not associate with disease progression [138]. Others have suggested that for IgAN, serum IgA1/C3 ratio may be a better marker for disease activity and progression than serum C3 levels alone [139,140]. Subsequently, Chen et al. investigated the relationship between the serum galactose-deficient IgA1/C3 ratio and disease progression in 1210 IgAN patients [141]. The galactose-deficient IgA1/C3 ratio had a much stronger association with disease progression than either marker alone, and the risk of kidney failure increased continuously with the ratio. These findings do not only show the potential of galactose-deficient IgA1/C3 ratios for risk assessment in IgAN, but also suggest that the complement-activating ability of the galactose-deficient IgA1 immune complexes determines disease severity. Terminal pathway activation leading to the generation of C5a and sC5b-9 has also been evaluated in IgAN, although much less extensively. A single study performed by Zwirner et al. found no differences in plasma sC5b-9 levels between patients with IgAN, Henoch–Schonlein purpura, and non-immune kidney disease [135]. In addition, none of the sC5b-9 values in IgAN patients exceeded the normal range, as defined by levels in the non-immune renal disease group. In a larger Taiwanese cohort, plasma levels of C5a were found to be higher in IgAN patients [87]. However, these patients were compared to healthy controls and patients with primary focal segmental sclerosis. Interestingly, IgAN patients who received immunosuppression had lower levels of C5a as early as 1 month after treatment.

Serologic evidence of alternative pathway activation (and/or the amplification loop) has also been documented in IgAN. Overall, IgAN patients seem to have higher systemic levels of alternative pathway components, as well as complement regulators [117,142]. Plasma levels of Ba, the smaller activation fragment of Factor B, were shown to be increased in IgAN patients compared to healthy controls and patients with primary focal segmental sclerosis [87]. Additionally, plasma Ba levels positively correlated with plasma levels of C5a levels, as well as weakly (yet statistically significantly) with the degree of proteinuria and impaired renal function. Recent work has investigated circulating levels of the FHRs in IgAN. Plasma levels of FHR-1 were shown to be elevated in Spanish IgAN patients compared to controls, whereas Factor H levels were normal [99]. In accordance, FHR-1/Factor H ratios were also elevated in IgAN, and the highest FHR-1 levels and FHR-1/Factor H ratios were found in patients with IgAN with disease progression. A separate study confirmed these results and demonstrated that plasma FHR-1 and the plasma FHR-1/Factor H ratio were increased in IgAN and associated with progression of the disease [100]. In addition, two independent studies showed that serum levels of FHR-5 were significantly higher in IgAN patients than in control patients [100,106]. In a British cohort, serum levels of FHR-5 were associated with more severe histology and unresponsiveness to immunosuppression, but not with progressive disease [100]. In a Chinese cohort, serum levels of FHR-5 were also associated with increased histological injury [106]. However, in contrast to the British cohort, Zhu et al. did report an association between serum FHR-5 levels and the risk of progressive disease. Whether these differences are due to dissimilar definitions of progressive disease or the consequence of ethnic/geographical differences remains to be determined. Nevertheless, these data, therefore, support the hypothesis that FHR-1 and FHR-5 compete with the regulatory function of Factor H. Factor H tips the balance towards alternative pathway inhibition and reduces the severity of the inflammatory injury, whereas these FHRs amplify alternative pathway activation and thereby stimulate IgAN development and progression of the disease (Table 1) [143]. 

Circulating levels of lectin pathway components have also been linked to IgAN severity. However, this association was complex and U-shaped, indicating that both low and high MBL levels associate with a higher risk, whereas IgAN patients with midrange levels are protected [144]. MBL deficiency in IgAN patients was associated with 50% loss of kidney function or kidney failure, whereas high levels of MBL (>3540 ng/mL) was associated with various markers of disease severity, including cellular crescents in the kidney biopsy and the degree of proteinuria, although the significance was lost after adjustment for other clinical variables. Furthermore, circulating levels of MBL do not seem to correlate with glomerular MBL deposits in the kidney biopsy [78]. Plasma levels of other lectin pathway components have also been investigated in IgAN. Circulating levels of ficolin-1, ficolin-2, MASP-1, and MBL-associated protein 2 (MAP-2) were increased in IgAN patients compared to healthy controls, but did not differ between IgAN patients with stable and progressive disease [117]. MAP-2 (previously MAp19) is an alternative splice product of the MASP-2 gene, and since this truncated form of 19 kDa lacks the serine protease domain, little is known about its function [145]. Earlier studies also reported systemic C4 activation in IgAN patients. Plasma C4d/C4 ratios, as a marker of C4 activation, were increased on at least one occasion in 28% of the adult IgAN patients [136]. Unfortunately, these studies have not been repeated since then. It would be especially interesting to see if plasma C4d levels in IgAN patients correlate with the extent of glomerular C4d deposits, since this has been demonstrated for other types of glomerulonephritis [146]. Initially, serum levels of C4bp were reported to be higher in IgAN patients than controls [90]. Others were not able to confirm these results, but did find that C4bp levels were higher in IgAN patients with worse prognoses [142]. Recently, Medjeral-Thomas et al. demonstrated that IgAN patients have reduced levels of MASP-3 compared to healthy controls [117]. Moreover, reduced MASP-3 levels were associated with the progression of IgAN [90]. These findings warrant further investigation, since MASP-3 is a vital player in the interaction between LP and AP and could clarify the connection between these two pathways in IgAN [25].

### 4.3. Genetic Variants in Complement Genes

Numerous studies support a strong genetic contribution to IgAN, and it was through these genetic studies that the concept of an autoimmune etiology originated [147]. Genome-wide association studies (GWAS) have revealed that disease susceptibility is greatly impacted by genetic variants in the antigen processing and presentation pathway, as well as the mucosal defense system [101,102,148]. Furthermore, GWAS highlighted the involvement of the complement system in IgAN [101,102,103]. These studies identified a common deletion within the Factor H gene locus as protective against IgAN (Table 1). This protective deletion results in the loss of the genes for FHR-3 and FHR-1 (CFHR3,1Δ) while leaving the gene for Factor H intact, and each copy of the deletion reduces the risk of IgAN by nearly 40% [101,103]. Interestingly, CFHR3,1Δ has been found with a relatively high prevalence, and the population frequency ranges from 0% in East Asians to 20% in Europeans, and up to 50% in certain African populations [149]. Moreover, CFHR3,1Δ has been associated with a lower risk for the development of AMD and IgAN, whereas it increases the risk for systemic lupus erythematosus (SLE) and aHUS (because of anti-Factor H autoantibodies) [101,150,151,152]. Fine mapping of the Factor H gene cluster in Chinese cohorts confirmed that CFHR3,1Δ is strongly protective against IgAN [104]. Furthermore, in IgAN patients, the deletion was associated with a lower prevalence of glomerular segmental sclerosis, tubular atrophy and interstitial fibrosis [104]. Further mechanistic studies revealed that CFHR3,1Δ in IgAN is associated with reduced mesangial C3 deposition and higher circulating levels of Factor H and C3, together with lower circulating C3a levels [153,154]. Recently, CFHR3,1Δ was also shown to be associated with better graft survival in patients who received a kidney transplant for IgAN [155]. In conclusion, the mechanism behind the protective effect of CFHR3,1Δ in IgAN is thought to arise from the reduced competition of FHRs with Factor H, thereby promoting inhibition rather than activation and accordingly reducing inflammation. In conformity, genetic variants of Factor H associated with lower plasma levels have also been identified in IgAN patients, suggesting that impaired regulation due to Factor H deficiencies could equally increase disease susceptibility [99]. Rare genetic variants of FHR-5 have also been described in IgAN, and allele frequencies differed significantly from that in controls [105]. The exact mechanism behind the association of these variants with IgAN remains unclear, but the FHR-5 variants are suggested to have increased binding capacity for C3b [105].

Genetics have also been utilized to advance the understanding of the lectin pathway in IgAN susceptibility and severity, especially for MBL. In the general population, there is a wide variation in circulating levels of MBL due to common genetic variants in the MBL gene (MBL2) [156]. The incidence of a MBL deficiency differs among populations, with the highest reported prevalence of more than 60% found in certain South American Indian groups [157]. The influence of MBL polymorphisms in IgAN was first investigated in a cohort of 77 IgAN patients and 140 controls [158]. Although no major conclusions could be drawn from this initial study, it is interesting to note that certain allele frequencies were lower in IgAN patients compared to controls. Conversely, Shi et al. found that IgAN patients with an MBL polymorphism in codon 54, which is associated with lower plasma levels, had a worse prognosis [159]. A separate study of Chinese patients investigated the impact of MBL2 gene polymorphisms on IgAN in a cohort of 749 IgAN patients and 489 controls [144]. The study found no differences in MBL2 haplotypes between IgAN patients and healthy controls, although a tendency was seen for a lower frequency of the O allele, which leads to a reduction in MBL functionality. These findings would suggest a protective role for low-producing MBL variants. Recently, the impact of MBL2 and ficolin-2 gene (FCN2) polymorphisms on disease progression were explored in over 1000 IgAN patients [160]. After screening for candidate variants through complete genetic sequencing of MBL2 and FCN2 in a small subset of patients, 7 expression-associated variations were further assessed in the discovery cohort. After adjustment for clinical and pathologic risk factors in multivariate analysis, only one variant in MBL2 (rs1800450) was associated with progression to kidney failure in IgAN patients. Moreover, the association remained significant in their validation cohort. The minor allele of rs1800450 G > A polymorphism was found to be associated with lower plasma levels of MBL, and homozygous IgAN patients had no detectable MBL levels, no glomerular deposition of MBL, increased histological injury as well as an increased risk of disease progression to kidney failure. Overall, the impact of MBL2 variants on IgAN can therefore not be unequivocally defined, since low-producing variants have both been suggested to be detrimental and beneficial.

## 5. Therapeutic Complement Inhibition in IgA Nephropathy

The growing body of evidence linking complement activation to the pathogenesis of IgAN has encouraged the study of complement-targeted therapies in this disease. To date, multiple clinical trials are ongoing to evaluate the safety and efficacy of different complement inhibitors in IgAN (Table 2). The targets of these therapies include MASP-2, C3, Factor B, C5, and C5aR1. Unfortunately, limited information has thus far been made available regarding these trials. The impressive panel of compounds currently pursued in IgAN is slightly surprising, since little data exist on preclinical complement inhibition in IgAN due to the lack of appropriate animal models. Zhang et al. demonstrated in a mouse model of IgAN that C3aR and C5aR1 deficiency leads to improved histology and reduced proteinuria [161]. These data, together with the fact that renal expression of C3aR and C5aR1 in IgAN patients correlates with disease activity and severity of renal injury, suggests that targeting C3aR or C5aR1 pharmaceutically could form a successful treatment option [132]. In accordance, preliminary data from the open-label phase II trial with avacopan, a C5aR1 antagonist, demonstrated reduced proteinuria and clinical improvement in 3 of the 7 IgAN patients (NCT02384317) [162]. In a Phase III trial involving patients with ANCA-associated vasculitis, avacopan was shown to be superior compared to prednisone in regards to remission rates, and the U.S. Food and Drug Administration (FDA) has approved avacopan as an adjunctive treatment for ANCA-associated vasculitis [163]. Effects of Eeulizumab treatment, a monoclonal antibody against C5, in IgAN were evaluated in two case reports as well as in a patient with IgAN recurrence after kidney donation with inconsistent results [164,165,166]. Nevertheless, ravulizumab, a long-acting anti-C5 blocking antibody engineered from eculizumab, is currently being evaluated in a Phase II trial for the treatment of IgAN (NCT04564339). Furthermore, small interfering RNA-targeting C5 (ALN-CC5) is also being evaluated in a Phase II trial in IgAN (NCT03841448). In addition to targeting the terminal pathway, inhibition of C3 with APL-2 is also being tested as a treatment option for IgAN (NCT03453619). APL-2 (pegcetacoplan) is a compstatin derivative that prevents C3 activation and has recently been approved by the FDA for the treatment of paroxysmal nocturnal hemoglobinuria (PNH) [167]. Efforts to specifically block activation of the alternative pathway (as well as the amplification loop) have led to the development of inhibitors that target Factor B. To this end, both Novartis and Ionis Pharmaceuticals are testing their Factor B inhibitors in phase II/III clinical trials in IgAN (NCT03373461, NCT04014335). The antisense Factor B inhibitor IONIS-FB-LRx (Ionis Pharmaceuticals) targets the production of Factor B, thereby effectively reducing circulating levels [168]. Meanwhile, Novartis has a small molecule inhibitor of Factor B that blocks the active site of Factor B and the Bb fragment [169]. From a different standpoint, targeting the lectin pathway through MASP-2 inhibition is also being pursued as a treatment option for IgAN. Blockage of MASP-2 would hamper glomerular lectin pathway activation, while still enabling C3 activation through the classical and alternative pathway. Narsoplimab (OMS721) is a humanized monoclonal antibody that blocks MASP-2; this antibody has been clinically developed by Omeros. Data of the phase II clinical trial with narsoplimab (OMS721) in IgAN were recently published [170]. First, 4 patients with corticosteroid-dependent IgAN were treated with 12 weekly infusions in a single-arm open-label substudy. After four weeks of initial Narsoplimab treatment, patients underwent steroid taper for the next four weeks, while the tapered steroid dose was maintained for the last four weeks. Next, these patients were followed up for six weeks after the last Narsoplimab infusion. Overall, the daily corticosteroid dose was reduced from 45 mg to 5 mg, and a median reduction of 72% was seen in 24-h urine protein excretion, while kidney function remained stable in all patients. Secondly, twelve patients with IgAN who were not receiving corticosteroids were randomized 1:1 to receive weekly narsoplimab infusions or vehicle for 12 weeks in a double-blind design. Once again, patients were followed for 6 weeks after the last treatment. After this follow-up period, all patients could enter dosing extension and receive narsoplimab. Overall reduction in proteinuria between the narsoplimab and vehicle groups was similar. However, for the eight patients that continued in the narsoplimab dosing extension (3 of which had initially received the vehicle), there was an overall decrease in proteinuria of 61.4%, suggesting a potential benefit. Interim analysis of both sub-studies indicated that the drug was safe and well-tolerated. Following up on these results, combined with a breakthrough therapy designation for IgAN by the FDA, the MASP-2 inhibitor is currently being tested in a Phase III, double-blind, randomized, and placebo-controlled study of IgAN patients with more than 1 g/day proteinuria (NCT03608033). A key unresolved question regarding the design of this trial remains whether MASP-2 inhibition will be equally effective in all IgAN patients, since histological lectin pathway activation is only seen in a subset of patients. 

## 6. Conclusions and Future Perspective

During the last few decades, a vast body of data has demonstrated the importance of the complement system, specifically the lectin and alternative pathway, as key drivers of pathology in IgAN. Complement activation has been shown to occur on circulating galactose-deficient IgA-immune complexes and in the glomerular mesangium after their deposition, thereby initiating and/or amplifying glomerular inflammation and kidney injury. Furthermore, acquired and inherited complement abnormalities that lead to complement dysregulation or a more active complement system alter disease susceptibility and the risk of progression. Despite these major advances, IgAN remains a challenging disease for physicians because of its heterogeneity and the risk to cause kidney failure. Complement measurements and histology for complement proteins could help to determine disease activity and severity. Additionally, this could enable personalized approaches by selecting patients for complement targeted therapies or other novel treatments. Multiple clinical trials with an impressive panel of complement inhibitors are currently ongoing, giving an exciting glimpse at the potential of using complement inhibitors for the treatment of IgAN. The approval of complement inhibitors for IgAN would be a major milestone for multiple reasons. It is also worth mentioning that IgAN could be the first common disease to be treated with complement inhibitors, since previous complement drugs have all been granted to rare and orphan diseases (e.g., aHUS and PNH). Moreover, any discussion of the use of complement inhibitors in patients with IgAN also needs to consider the costs. The excessive costs of current complement inhibitors, such as eculizumab, cannot be overlooked (approximately $500,000 per year per patient). Such pricing may be acceptable for rare indications, but not for a common disease such as IgAN. However, lower drug pricing could be achieved by extending the applications of complement-targeted therapies to a larger patient population, such as those with IgAN or other forms of glomerulonephritis. 

## Figures and Tables

**Table 1:** The role of the Factor H protein family in IgA nephropathy.

	Evidence for the Involvement of the Factor H Protein Family in the Pathogenesis of IgA Nephropathy		
	Genetic	Histologic	Serologic
Factor H	Genetic variants of Factor H associated with lower plasma levels may contribute to genetic susceptibility to IgAN [99].	Glomerular deposition of Factor H staining is reduced in patients with progressive IgAN compared to stable disease. Absence of glomerular Factor H deposition is associated with progressive disease [88].	Plasma Factor H levels are not altered in IgAN patients, and these levels are not associated with disease severity, but the plasma FHR-1/Factor H ratio is associated with disease progression [99,100].
Factor H-related protein 1 (FHR-1)	The deletion of complement factor H-related proteins 3 and 1 genes (CFHR3,1Δ) is associated with protection against IgAN [101,102,103,104].	Proteomics showed that FHR-1 is more abundant in the glomeruli of IgAN patients compared to controls. Glomerular FHR-1 deposits have also been identified in IgAN, but no association is seen with IgAN severity [88,96].	Plasma FHR-1 levels are elevated in IgAN patients compared to healthy controls, and the plasma FHR-1/Factor H ratio is associated with disease progression of the disease [99,100].
Factor H-related protein 2 (FHR-2)	N.D.	Proteomic analysis revealed that FHR-2 is more abundant in the glomeruli of patients with progressive IgAN compared to non-progressive IgAN [96].	N.D.
Factor H-related protein 3 (FHR-3)	The deletion of complement factor H-related proteins 3 and 1 genes (CFHR3,1Δ) is associated with protection against IgAN [101,102,103,104].	Proteomic analysis demonstrated that FHR-3 is more abundant in the glomeruli of IgAN patients compared to controls [96].	N.D.
Factor H-related protein 4 (FHR-4)	N.D.	N.D.	N.D.
Factor H-related protein 5 (FHR-5)	Rare genetic variants in FHR-5 may contribute to the genetic susceptibility to IgAN [105].	Glomerular FHR-5 deposits have been identified in IgAN and correlate with C3 and C5b-9 deposits. Increased glomerular staining for FHR-5 is associated with more severe histology and progressive disease [88,96,97,98].	Serum FHR-5 levels are higher in IgAN patients compared to healthy controls and are associated with more severe histology, unresponsiveness to immunosuppression, and disease progression [100,106].

An overview of all the available evidence of the involvement of the Factor H protein family in IgA nephropathy. Abbreviations: N.D, not determined; IgAN, IgA Nephropathy; FHR-1, Factor H-related protein 1; FHR-2, Factor H-related protein 2; FHR-3, Factor H-related protein 3; FHR-4, Factor H-related protein 4; FHR-5, Factor H-related protein 5.

**Table 2:** Clinical trials with complement inhibitors in IgA nephropathy.

Trail ID	Target	Compound	Company	Design	Status
NCT03608033	MASP-2	Monoclonal antibody, intravenous injection	Omeros	Randomized, double-blind, placebo-controlled, Phase 3 study	Ongoing
NCT03453619	C3	Pegylated peptide, subcutaneous injection	Apellis Pharmaceuticals	Single arm open-label Phase 2 study	Ongoing
NCT04578834	Factor B	Small molecule, orally administered	Novartis	Multi-center, randomized, double-blind, placebo-controlled, Phase 3 study	Ongoing
NCT04014335	Factor B	Antisense oligonucleotide, subcutaneous injection	Ionis Pharmaceuticals	Single arm open-label Phase 2 study	Ongoing
NCT04564339	C5	Monoclonal antibody, intravenous injection	Alexion Pharmaceuticals	Randomized, double-blind, placebo-controlled Phase 2 study	Ongoing
NCT03841448	C5	Small interfering RNA, subcutaneous injection	Alnylam Pharmaceuticals	Randomized, double-blind, placebo-controlled Phase 2 study	Ongoing
NCT02384317	C5aR1	Small molecule, orally administered	Chemocentryx	Single arm open-label Phase 2 study	Completed

An overview of complement inhibitors that are currently being evaluated in clinical trials of IgA nephropathy. Last updated on 1 September 2021. Abbreviations: C5aR1, C5a receptor 1; MASP-2, mannose-binding protein-associated serine protease 2.
